# Monitoring soil radon during the 2016–2017 central Italy sequence in light of seismicity

**DOI:** 10.1038/s41598-020-69821-2

**Published:** 2020-08-04

**Authors:** Gaia Soldati, Valentina Cannelli, Antonio Piersanti

**Affiliations:** Istituto Nazionale di Geofisica e Vulcanologia, via di Vigna Murata 605, 00143 Rome, Italy

**Keywords:** Geochemistry, Geophysics, Seismology

## Abstract

The radioactive nature of radon makes it a powerful tracer for fluid movements in the crust, and a potentially effective marker to study processes connected with earthquakes preparatory phase. To explore the feasibility of using soil radon variations as earthquakes precursor, we analyse the radon concentration data recorded by two stations located close to the epicentre of the strongest mainshock (Mw 6.5 on October 30, 2016) of the seismic sequence which affected central Italy from August 2016. The two stations CTTR and NRCA operate in the framework of the permanent Italian Radon monitoring Network and recorded almost continuously since 2012 and 2016, respectively, the latter being installed just after the first mainshock of the sequence (Mw 6.0 on August 24, 2016). An increase of radon emanation is clearly visible about 2 weeks before the Mw 6.5 event on both the time series, more pronounced on NRCA, nearer to the epicentre, suggesting the possibility of a direct association with the earthquake occurrence. An independently developed detection algorithm aimed at highlighting the connections between radon emission variations and major earthquakes occurrence succeeds in forecasting the Mw 6.5 mainshock on NRCA time series. The resulting time advance of the alarm is consistent with that obtained using a Bayesian approach to compute the a posteriori probability of multiple change points on the radon time series of NRCA. Moreover, it is in agreement with the delay time which maximizes the correlation between radon and seismic anomalies. Applying the detection algorithm to CTTR time series returns alarms for both the Mw 6.0 event, with epicentre closer to this station, and the stronger Mw 6.5 event, but with a higher number of false detections. Finally, we found that a preliminary correction of the bias introduced by variations of meteorological parameters does not affect our main finding of an increase in radon concentration before the major mainshocks. Our study confirms that, although much work is still needed, a monitoring approach based on a permanent dense network is crucial for making radon time series analysis an effective complement to traditional seismological tools.

## Introduction

**Figure 1:**
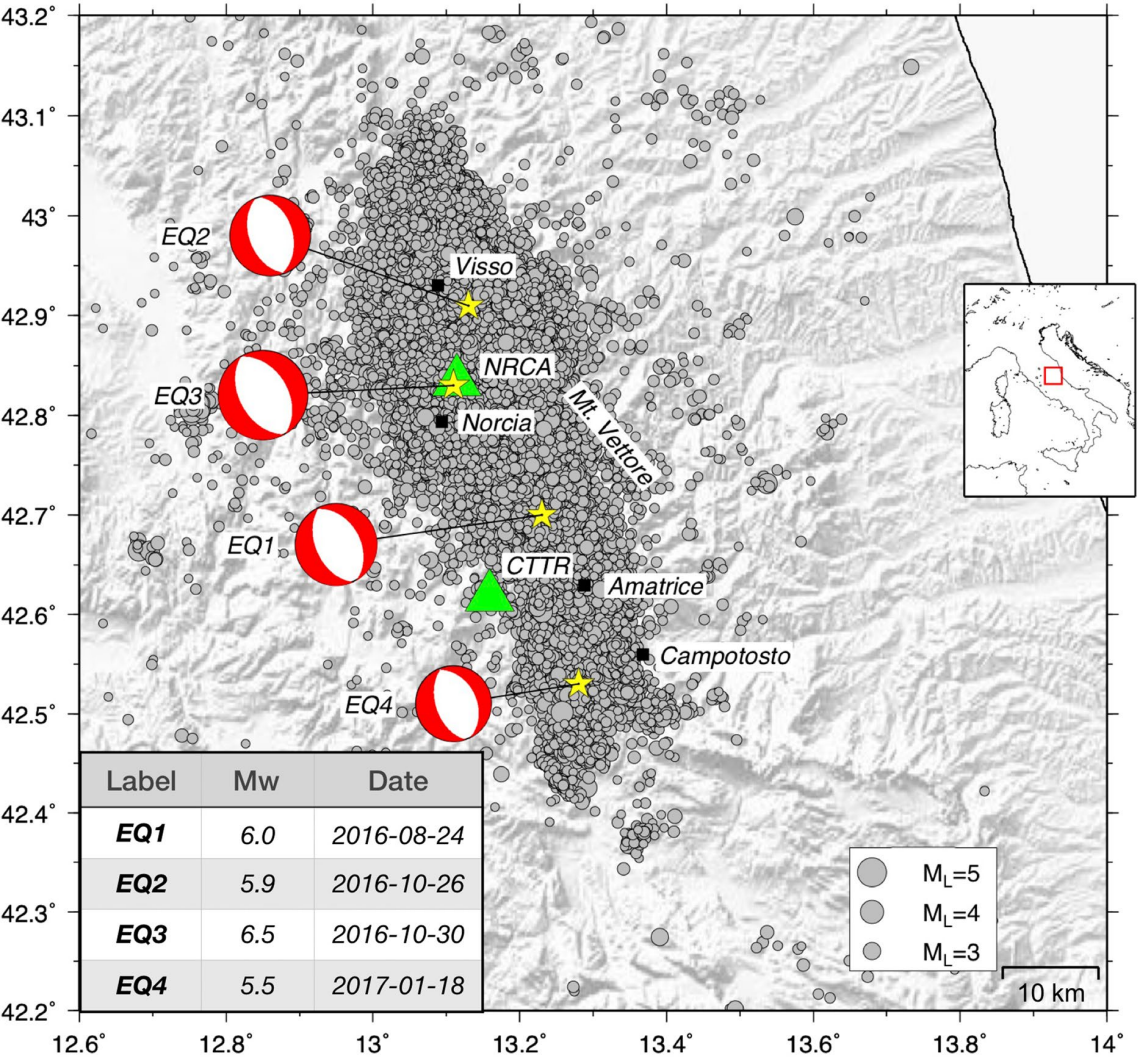
Geographical setting of the Amatrice-Visso-Norcia seismic sequence. Green triangles indicate the location of the radon monitoring stations CTTR and NRCA. The earthquakes with M ≥ 1.5 recorded by the INSN from July 2016 to February 2017 (about 37,000 events) are shown as grey circles. Yellow stars indicate the epicentres (along with corresponding focal mechanisms) of the 4 largest mainshocks of the sequence, labeled according to the inset table on the bottom left corner. The Generic Mapping Tools^30^, Version 5.2.1, (https://gmt.soest.hawaii.edu/) were used to generate the map.

Since the end of August 2016, a seismic sequence—later named “Amatrice-Visso-Norcia”—affected a large portion of central Italy, with a number of recorded events exceeding 110,000 over 3 years (catalogue of the Italian National Seismic Network^1^ (INSN, https://www.fdsn.org/networks/detail/IV/)). The sequence started on August 24, 2016 with a Mw 6.0 earthquake (hereinafter, EQ1) at Accumoli (Amatrice), followed on October 26 by two events of Mw 5.4 and Mw 5.9 (EQ2) located around Visso, about 25 km NW of EQ1’s epicentre. 4 days later a Mw 6.5 earthquake (EQ3) nucleated in the vicinity of Norcia, nearly 10 km S of Visso (see Fig. 1). After that, the seismicity started to slowly decrease, with the exception of the 4 Mw > 5 earthquakes (EQ4) further S, near Montereale (Campotosto), on January 18, 2017. Currently (November 2019), the cumulative daily release of seismic moment is still higher than the pre-earthquake level of July 2016.

The main events show NW–SE striking normal faulting, in agreement with the extensional tectonic regime of the central Apennines^2^, and activated an area with extension of almost 80 km along the Apennine belt. The estimated damage exceeds 23 billion euros and the life loss amounts to 300 fatalities. In terms of seismic energy and destructive impact, this sequence can be directly compared to the 1980 Irpinia (southern Italy) one. More details on the sequence may be found on a special issue (and references therein) of Annals of Geophysics^3^, and on up-to-date scientific reports published on the website https://www.ingv.it.

At about 11 km SW of EQ1 epicentre, a permanent radon monitoring station (CTTR) belonging to the Italian Radon mOnitoring Network^4^ (IRON) has been recording radon concentration data since the second half of 2010. Data acquired by this station until the end of August 2016 were analysed^5^, along with the outcome of laboratory experiments on radon emission dynamics from rock samples subject to normal and shear stress loads. The study^5^ suggests that fluid migration could have played a minor role in the onset of the Amatrice seismic event with respect to previous Apennine earthquake sequences (e.g. Colfiorito 1997 and L’ Aquila 2009).

Right after the August 24, 2016 earthquake, a new station (NRCA) was co-located with the homonymous seismic one of the Italian National Seismic Network (INGV-INSN), nearly 5 km NE of the epicentre of the forthcoming EQ3, and it is continuously recording since then.

The availability of continuous, long-term soil radon measurements from two stations close to the location of the major earthquakes of the sequence represents an unprecedented chance to study radon transient signals potentially related to the temporal evolution of seismicity.

Thanks to its radioactive nature, radon is considered a powerful tracer for fluid movements in the crust, thus a proxy to study processes connected with the preparatory phase of earthquakes^6^. Indeed, radioactive detectors are among the most sensitive instruments since their efficiency in detecting and measuring ionizing radiation is far higher than any other non-radioactive element-detecting instrument^7,8^.

A link between the rock state of stress and variations in the radon emanation properties has been also suggested and confirmed by several laboratory experiments^9–11^.

The main limitation, when trying to associate soil radon variations with Earth’s internal processes, is the difficulty to identify those due to environmental effects, such as meteorological parameters and/or random noise^6^. In fact, seismo-tectonically and meteorologically induced signals in radon time series may look very similar. The identification of the multiple, non-linearly interacting factors affecting radon release from source and transfer to the surface is a key aspect for all radon applications.

Meteorological conditions are thought to be a major influence on radon migration, since rainfall, winds, and surface temperature variations induce micro-pressure gradients and convective dynamics that affects radon transportation in porous media, with typical characteristic times ranging from hours to a year^12,13^.

In the next section we describe how we collected the data of radon concentration and what emerges from a simple visual inspection of the raw time series. An empirical correction procedure^14^, originally developed, is then applied in order to try to limit the bias induced by variations of the meteorological parameters. We then test the performance of a real time detection algorithm in highlighting the connections between radon emission variations and the occurrence of major seismic events. The results are cross-checked by means of a change point analysis that uses a Bayesian approach to calculate the posterior probability of multiple change points in a generic time series. Finally, the cross-correlation between time series of radon concentration and seismic moment release is performed as a further check of the delay time between radon and seismic anomalies resulting from the analyses above.

## Method and results

### Data

Stations CTTR and NRCA are equipped with a high sensitivity active radon monitoring instrument based on a Lucas cell^15^, with high efficiency (0.06 and 0.07 count min^−1^/Bq m^−3^, respectively) and minimum detectable concentration of 6 Bq m^−3^. The acquisition time is set to 115 min, and the stations acquire simultaneously local temperature values by means of a specific sensor. The daily values of external temperature, pressure, and precipitation are provided by the weather website https://www.ilmeteo.it as short term (12–24 h) forecasts. Figure S2 of the Supplementary material displays the available meteorological observables (temperature, pressure, rainfall) along with the radon time series collected at the two stations.

While the instrument at CTTR is located in a basement hosting the municipal archive of the city occasionally accessible only to technical staff (indoor installation type), the one at NRCA is co-located in a small shelter (shelter installation type) with a seismic station belonging to the INSN monitoring network. Systematic analyses of time series recorded by IRON stations indicated that indoor installations are noisier than shelter ones and show larger variability and stronger seasonal signal, likely connected with temperature fluctuations^4^.

**Figure 2:**
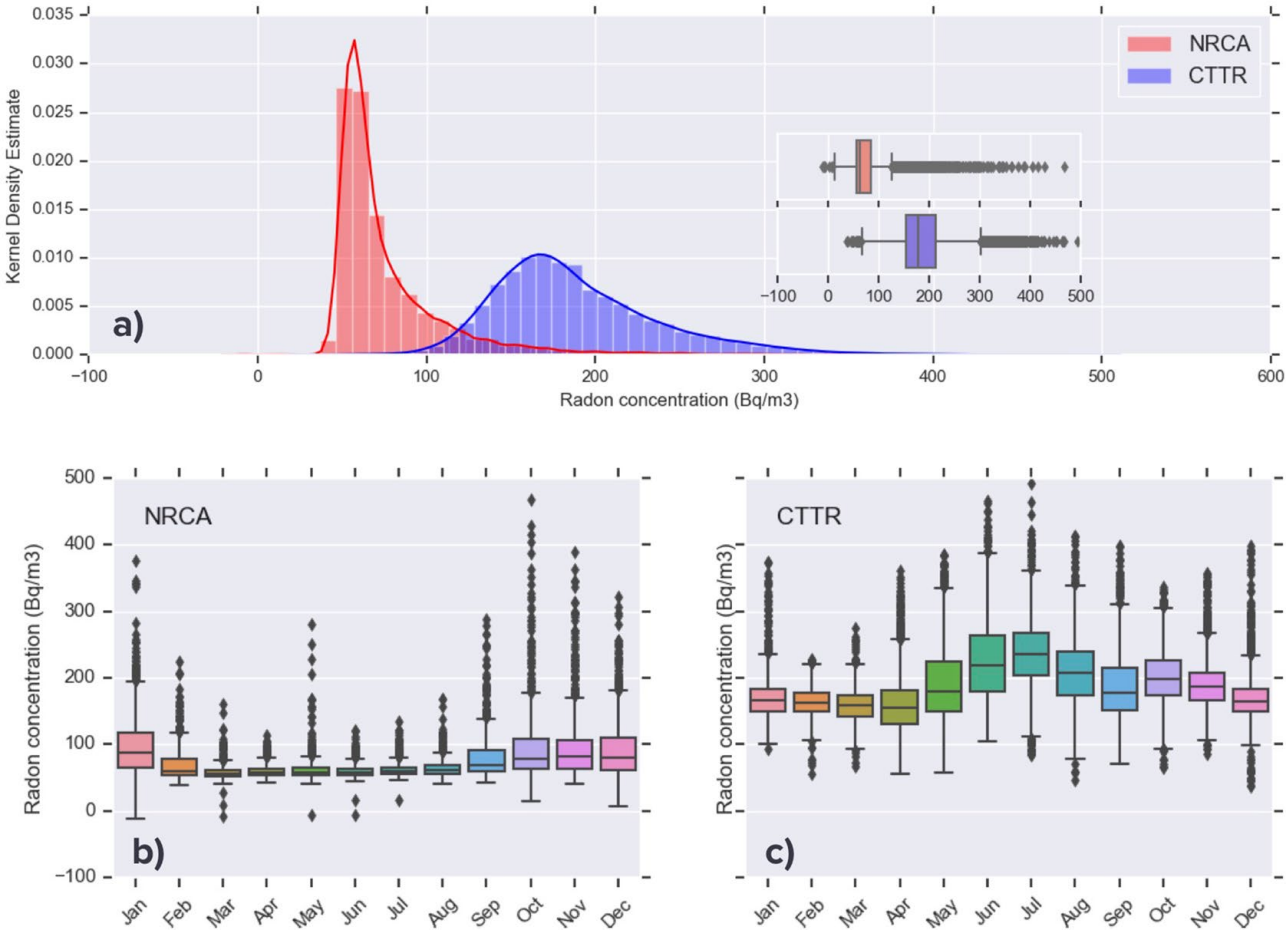
Estimated probability density function (kernel density estimate) and corresponding histogram for soil radon concentration data (**a**) collected at station NRCA (red) and CTTR (blue). (**b**,**c**) Month-wise boxplot of the same data.

This is confirmed by Fig. 2, showing for the two stations considered the distribution of radon concentration data through a density plot and a boxplot. The probability density function (Fig. 2a) estimated using Gaussian kernels^16^ indicates that the dataset recorded by the indoor station CTTR (blue) shows larger dispersion with respect to the NRCA one (red). The boxplots in the inset summarise the main parameters describing the statistics of the two datasets: CTTR data are more scattered, with a larger central value (median) and a smaller degree of right skewness compared to NRCA data. Figure 2b,c, where radon data from the two stations are grouped at month intervals, evidence that soil radon emission at station NRCA is maximum in the cold season (October-January) while the opposite holds true for station CTTR, where radon variations are more correlated with air temperature and more scattered during the whole year and markedly in the warm months. This seasonal periodicity has been also observed in laboratory tests^17^ and in several long term radon monitoring studies^18–20^.

Despite station CTTR has been recording almost continuously since July 2010, we selected here only the data collected since August 2012, when a new radon Lucas cell based detector was installed, for a total length of the time series of more than 7 years (2,367 days, 26,909 observations). Station NRCA was installed the day right after the occurrence of the 2016 Mw 6.0 Amatrice mainshock, so its data time series is less than 3-year long (1,042 days, 10,739 observations).

**Figure 3:**
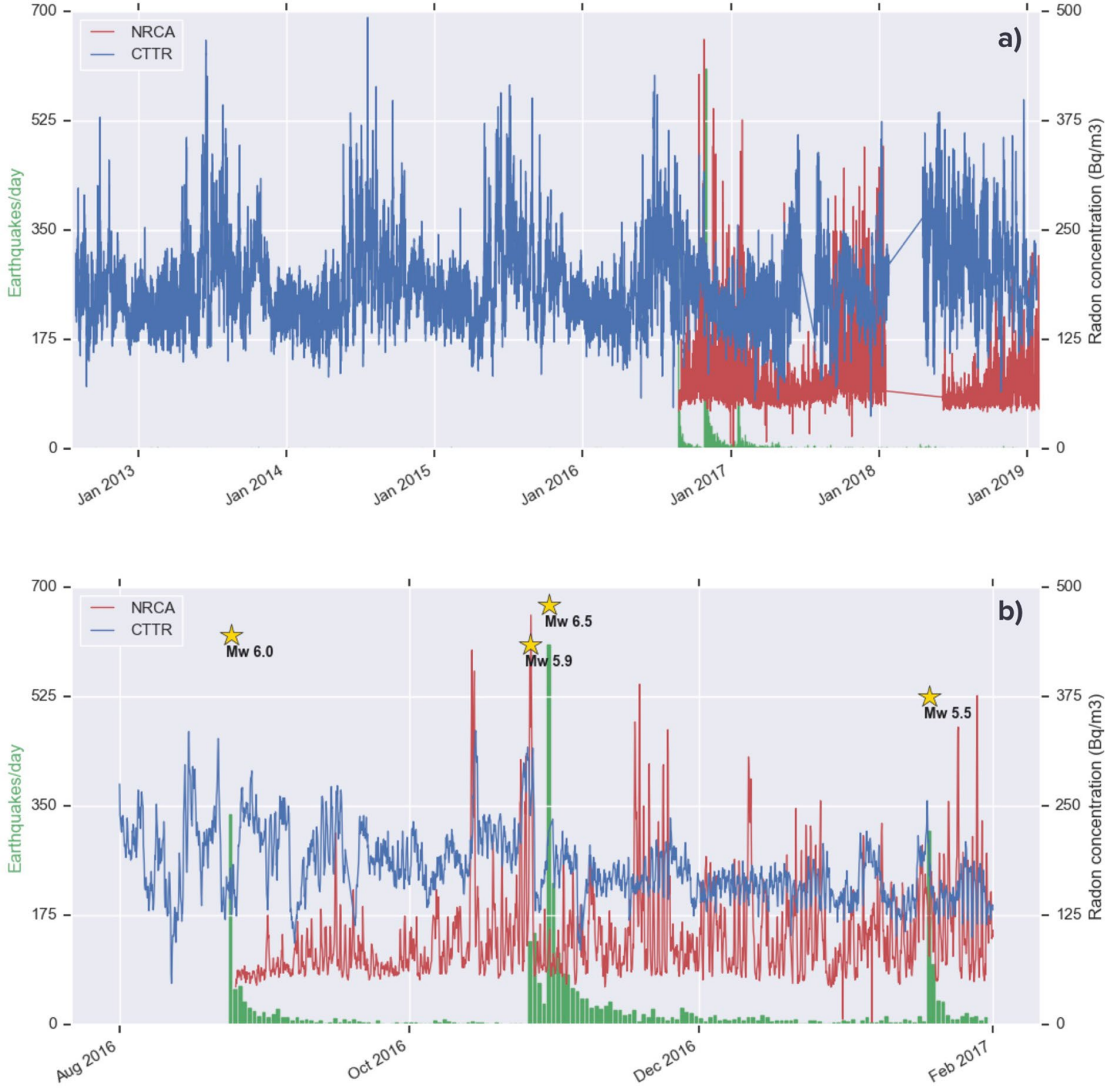
(**a**) Time series of soil radon concentration (Bq m^−3^) from NRCA (red) and CTTR (blue) stations. While CTTR registered almost continuously since August 2012, NRCA was installed the day right after the occurrence of the Amatrice Mw 6.0 mainshock. (**b**) Zoom over the period corresponding to the major seismic activity of the central Italy sequence of 2016–2017. Green bars indicate the number of earthquakes/day occurred within a radius of 40 km from the two stations, with yellow stars in correspondence of the four Mw ≥ 5.5 mainshocks (see Fig. 1).

The time series of raw radon concentration data (Bq m^−3^) of the stations NRCA and CTTR are shown in Fig. 3a in red and blue, respectively. The longer time series of station CTTR shows a marked seasonal signal, less evident on NRCA one. Together with the different installation type, this is also due to the limited length of NRCA time series and by the presence of gaps in the measurements. A zoom on the time corresponding to the major seismic activity of the central Italy sequence (Fig. 3b) shows an apparently decreasing trend on CTTR data, possibly an effect of the seasonal behaviour. Among the various peaks in NRCA radon concentration, the two major ones are shared with CTTR time series and are therefore presumed to be significant. They precede the occurrence of EQ3, and are more pronounced (both in absolute value and relative to the background signal) in NRCA time series. The first radon concentration peak occurs 2 weeks before EQ3 and the second one occurs on the same day of EQ2.

### Meteorological effects

Despite the wide literature on the subject^18–25^, a complete assessment of the relation between radon variations of tectonic origin and those due to atmospheric variables has not been established yet. In fact, the several studies addressing this question obtained results not univocal and often remarkably in contrast to each other. It is likely that the disagreement does not only reflect unreliability or weakness in the single analyses, but rather the strong site-dependency of the environmental effects, drastically influenced by the local features of the station site and by the installation typology.

A confirmation of this comes from the recent work by Siino et al. 2019, who carried on one the very few investigations in literature comparing as much as a dozen of concurrent radon time series widely distributed on a regional scale. The main outcome of this analysis, based on multivariate techniques, is that local site effects play a major role in modulating radon signal and, consequently, atmospheric correction approaches may not grant robust results.

Nevertheless, we make an attempt to employ an original empirical procedure^14^ aimed to limit the impact of environmental conditions on the interpretation of measured radon levels. The idea is to negatively weight the radon signal when it is associated to a peak of a meteorological parameter positively correlated with it, and to positively weight the radon signal if the meteorological parameter is negatively correlated with it.

To date, rainfall is the only meteorological parameter whose anti-correlation with radon data (due to the ‘ground sealing’ effect) is significant and widely observed. Conversely, the influence of temperature is strictly site-dependent, as it happens for the two stations considered here, which show opposite correlation with temperature variations (Figs. 2, 3). This is why at this stage of the analysis we chose to only take into account the effect of precipitation, leaving the treatment of the temperature- and pressure-induce bias to a future and more comprehensive study.

**Figure 4:**
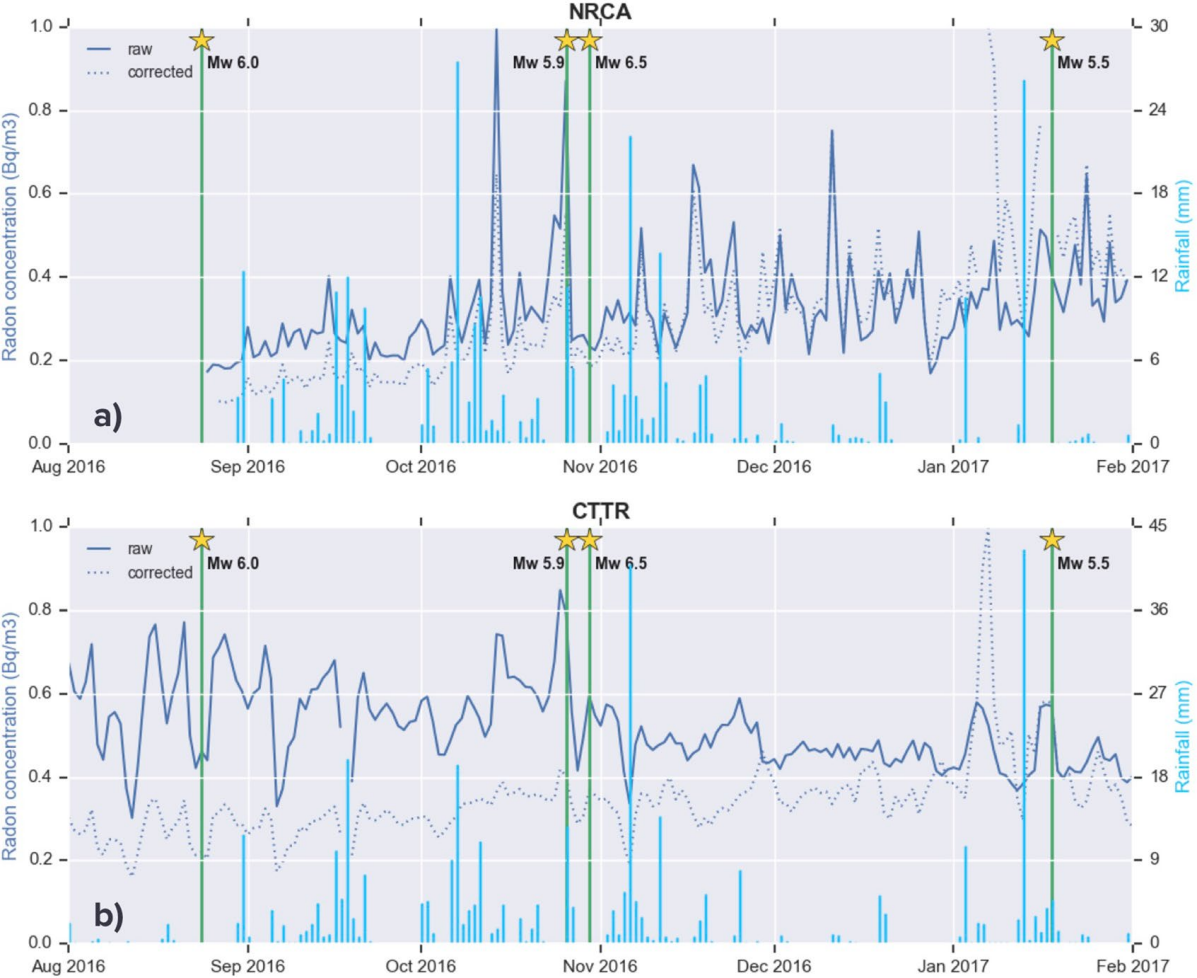
Time series of daily-averaged soil radon concentration (Bq m^−3^) from NRCA (top) and CTTR (bottom) stations, from August 2016 to February 2017. Solid and dashed blue lines indicate raw data and data corrected for the effect of precipitations (mm, light blue bars), respectively. Green lines represent the time of occurence of the four Mw ≥ 5.5 earthquakes (yellow stars) of the central Italy seismic sequence.

In Fig. 4, the raw and rainfall-corrected radon data, normalized according to their maximum value, are indicated in blue solid and dotted line, respectively. Green vertical lines indicate the time of occurrence of the four mainshocks and light-blue bars represent the daily amount of rainfall (mm). When the empirical correction is applied, the radon peak observed in raw time series on 15 October 2016 reduces of 50% in NRCA and becomes barely visible in CTTR, while the peak of 26 October still holds after the correction, meaning that it is presumably not associated with precipitation and rather to a deeper source common to the two stations.

### Detection algorithm: approach

The visual inspection of the time series of radon concentration may provide some first order observations on radon emanation features, but the detection of more subtle changes in the time series for predictive purposes requires more advanced tools. We employ an original detection algorithm^14^ (DA hereinafter), which basically checks whether the radon daily average increases over a given threshold and if this condition persists over time.

The DA is aimed to highlight potential connections between radon emission variations and the occurrence of major seismic events. It might in principle be used in real time analyses since it explores the available data time series to forecast future seismic events issuing alarms on the basis of significant variations of radon emanation.

Piersanti et al. 2016 present in their Figure 10a flowchart of the algorithm operation: when the radon daily average exceeds the moving average (computed on p1 days) by a factor (p2) on the day i and the radon moving average successively increases by a factor (p3) for a time window (w = p4*p1 days), an alarm is issued at day i + w. If an earthquake of magnitude larger than 4 occurs within 40 days from the date of the alarm, the parameter p2 is increased by a factor p5 for a time window proportional to the earthquake moment. After that time, in case of no further M > 4 earthquake, the parameter p1 is restored to its original value.

**Figure 5:**
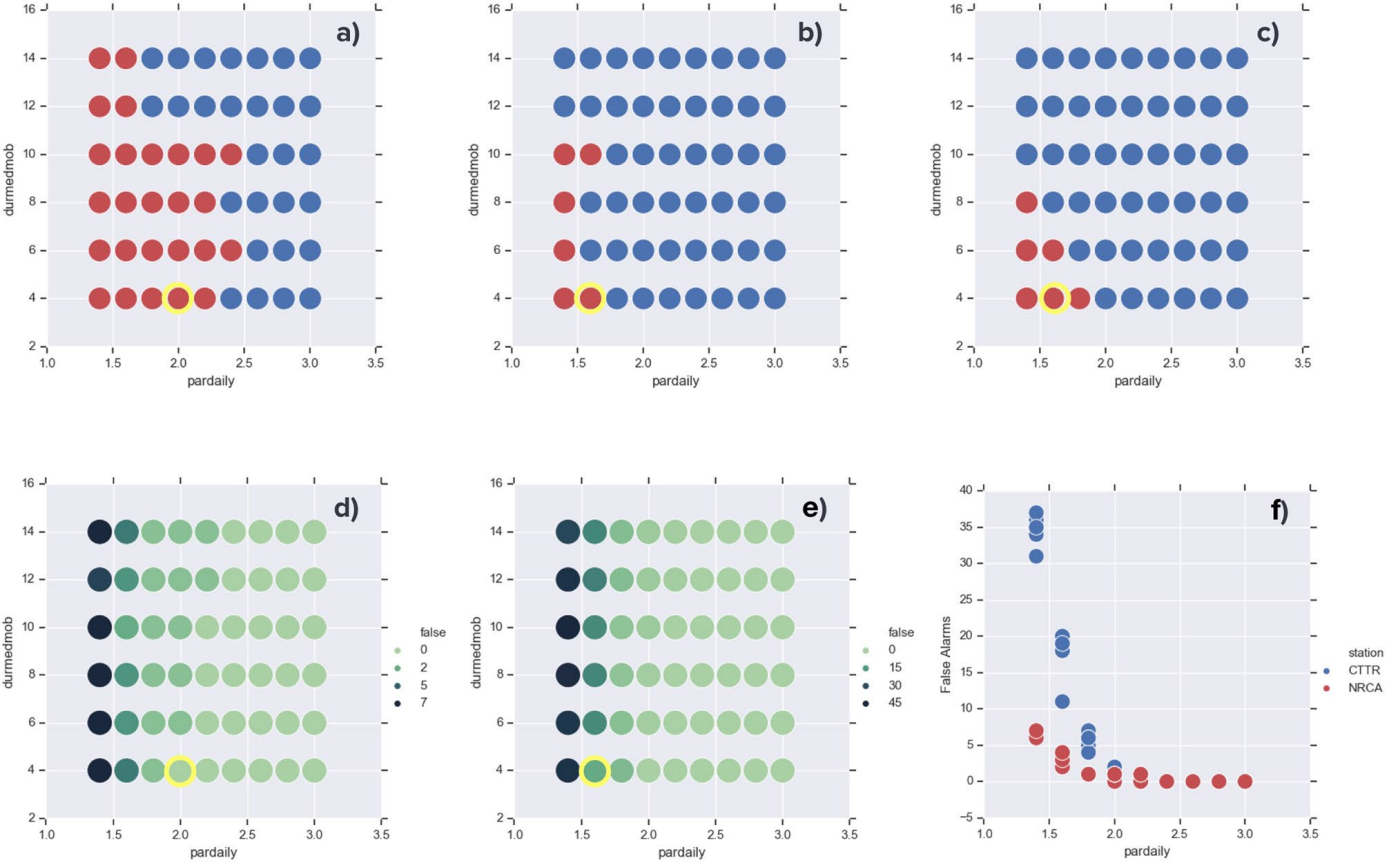
Effect of the parameters involved in the Detection Algorithm on the issued alarms preceding a given earthquake. (**a**,**b**) refer to radon time series of the stations NRCA and CTTR, respectively, and to the Mw 6.5 EQ3. Red/blue circles indicate if the alarm before that earthquake is issued/not issued. The combination of parameters durmedmob/pardaily (p1/p2) selected is highlighted in yellow. (**c**) refers to station CTTR and to the alarm preceding the Mw 6 EQ1. (**d**,**e**) Number of false alarms delivered running the algorithm on radon data from stations NRCA and CTTR, respectively, with the input parameters selected above. (**f**) dependence of false alarms on the input parameter pardaily (p2).

Figure 5 illustrates how the tuning of the main input parameters (p1 and p2) affects the output of the algorithm. Red/blue circles indicate whether or not an alarm before EQ3 is issued running the DA on the radon time series of the stations NRCA (a) and CTTR (b). The number of combinations of the parameters that obtains an alarm are quite reduced in the CTTR case with respect to the NRCA one, meaning that the earthquake is more difficult to detect on that time series. This is likely due to the larger station-epicentre distance and to the lower SNR. When that distance is reduced, as for station CTTR relative to EQ1, the alarm before this earthquake is issued also for higher value of p2 (Fig. 5c).

In view of a real time application of the algorithm, another important factor we consider to select the set of input parameters for the DA is the number of false alarms delivered. Figure 5d,e, corresponding to NRCA and CTTR radon data, demonstrate that while the false alarms markedly depend on p2 (pardaily), they are only slightly affected by the value of p1 (durmedmob). Figure 5f reveals the higher number of false alarms issued for the CTTR time series compared to NRCA one, under the same value of p2. This confirms that clean and less noisy data are more suitable for the detection of tectonic signals.

The combination of parameters p1-p2 selected is highlighted in yellow and represents the tradeoff between the highest detection threshold p2 for which the DA finds an alarm before the earthquakes EQ1 and EQ3, and the minimum number of false alarms delivered. The whole set of input parameters is displayed in Supplementary Table S1.

### Detection algorithm: results

**Figure 6:**
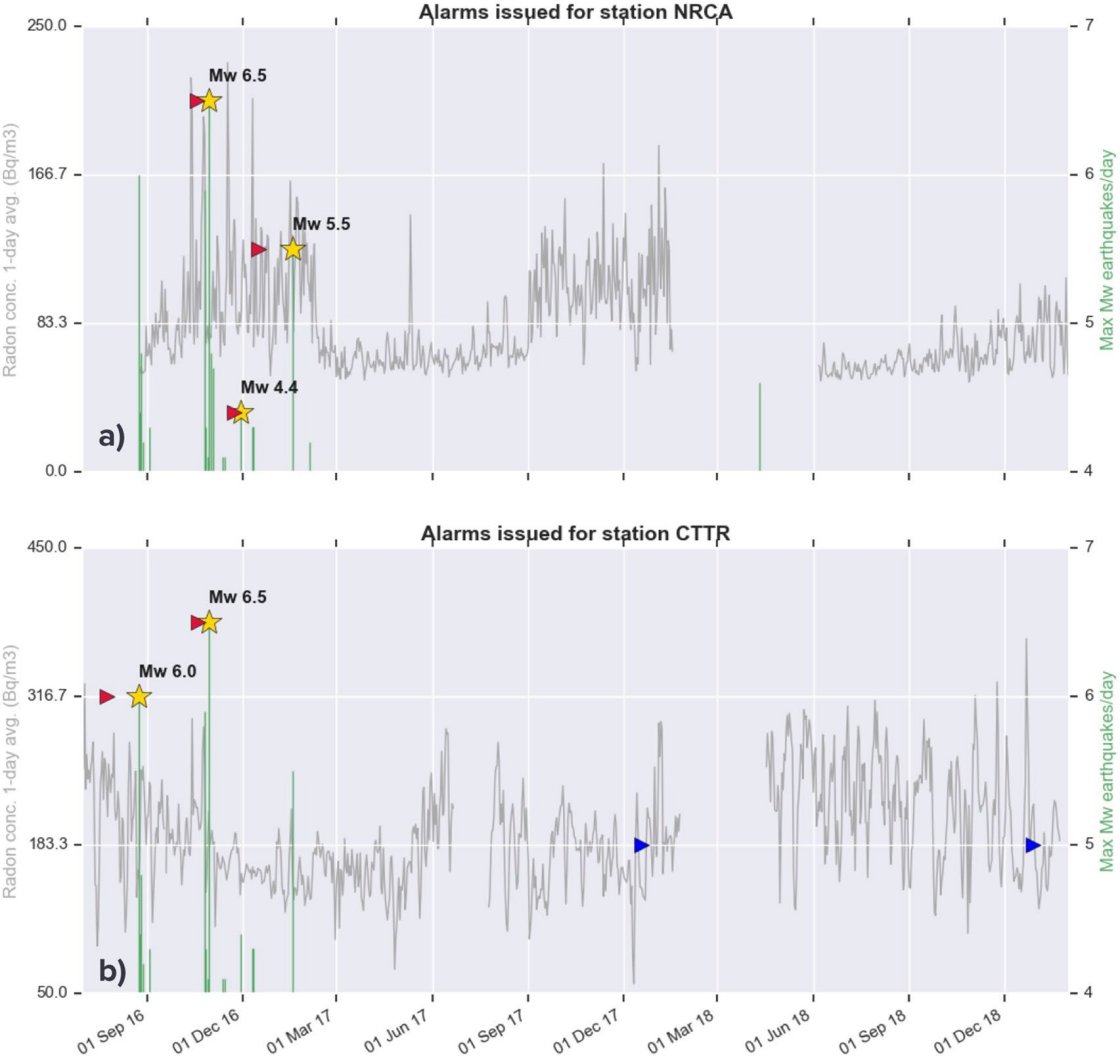
Alarms issued by the detection algorithm applied to the time series of radon concentration of NRCA (**a**) and CTTR (**b**), from the beginning of the central Italy seismic sequence. Alarms are considered true (red triangles) if followed within 40 days by a seismic event (yellow stars) with magnitude Mw ≥ 4.0, otherwise they are regarded as false (blue triangles).

Figure 6 shows the results of the DA applied to the time series of daily radon concentration (gray line) of NRCA (a) and CTTR stations (b). Green bars indicate the magnitude of the largest earthquake of the day occurred within a radius of 40 km from the station considered. The triangles represent all the issued alarms: in red the ones followed within 40 days by a Mw > 4 earthquake (yellow stars), in blue the remaining ones, deemed as ‘false’.

The DA applied to the NRCA time series successfully forecasts EQ3 and EQ4, along with a moderate-magnitude (Mw 4.4) earthquake preceding this last one. The first alarm is issued about 2 weeks before EQ3, a time advance consistent with the one characterizing the radon peak observed in NRCA and CTTR raw data (Fig. 3). We cannot rule out the hypothesis that the alarm points instead towards EQ2, occurred 4 days before EQ3.

**Figure 7:**
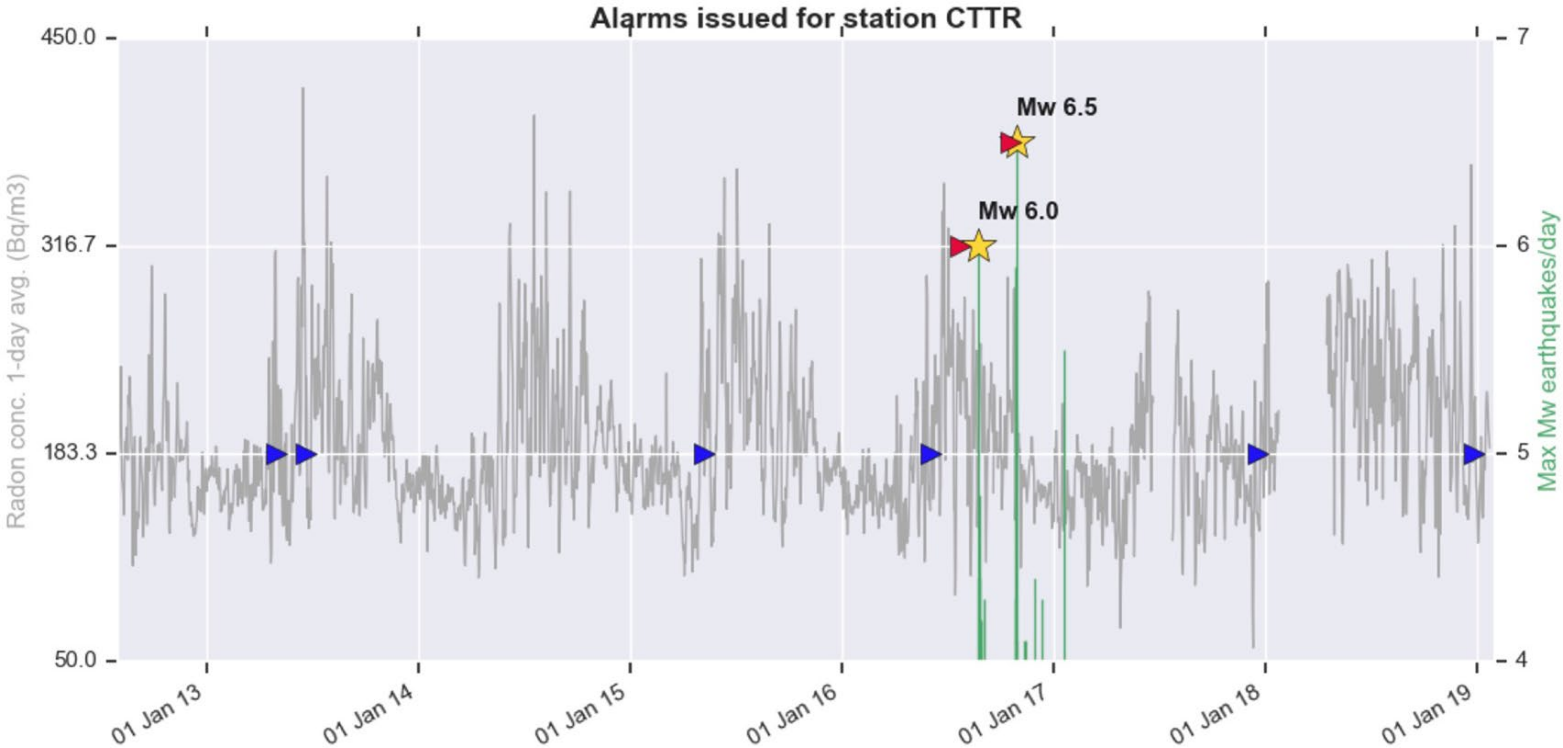
Same as previous figure, with the detection algorithm applied to the whole time series of radon concentration of CTTR station.

As for the CTTR times series, despite the higher noise (Fig. 2), the DA succeeds in forecasting both EQ1, whose epicentre is closer than the one of EQ3 to this station, and EQ3, more far away but with larger magnitude. It also delivers 2 false alarms within the end of 2018, which rise to 6 when the DA analysis is extended to the whole time series of CTTR station (Fig. 7), for a total of less than one alarm/year.

These false alarms show a likely seasonal origin, being issued mainly in May/June or November/December, i.e. at the beginning or at the end of the ‘warm’ season, when radon emanation is observed to be larger at station CTTR. We performed a series of tests aimed to remove the annual periodicity of the radon data by subtracting the sinusoid which better fits the data, by differencing, by decomposition of the series (as a sum of trend, seasonality, and residual), by filtering out low frequencies (1 year) by means of the Fast Fourier Transform. After running the DA on the time series so processed, no alarm is issued at all (neither true or false). We interpret this result as an indication that the variability of radon is similar to the one of temperature, and since the signal is comparable to the noise, the detrending techniques end with cancelling out both.

**Figure 8:**
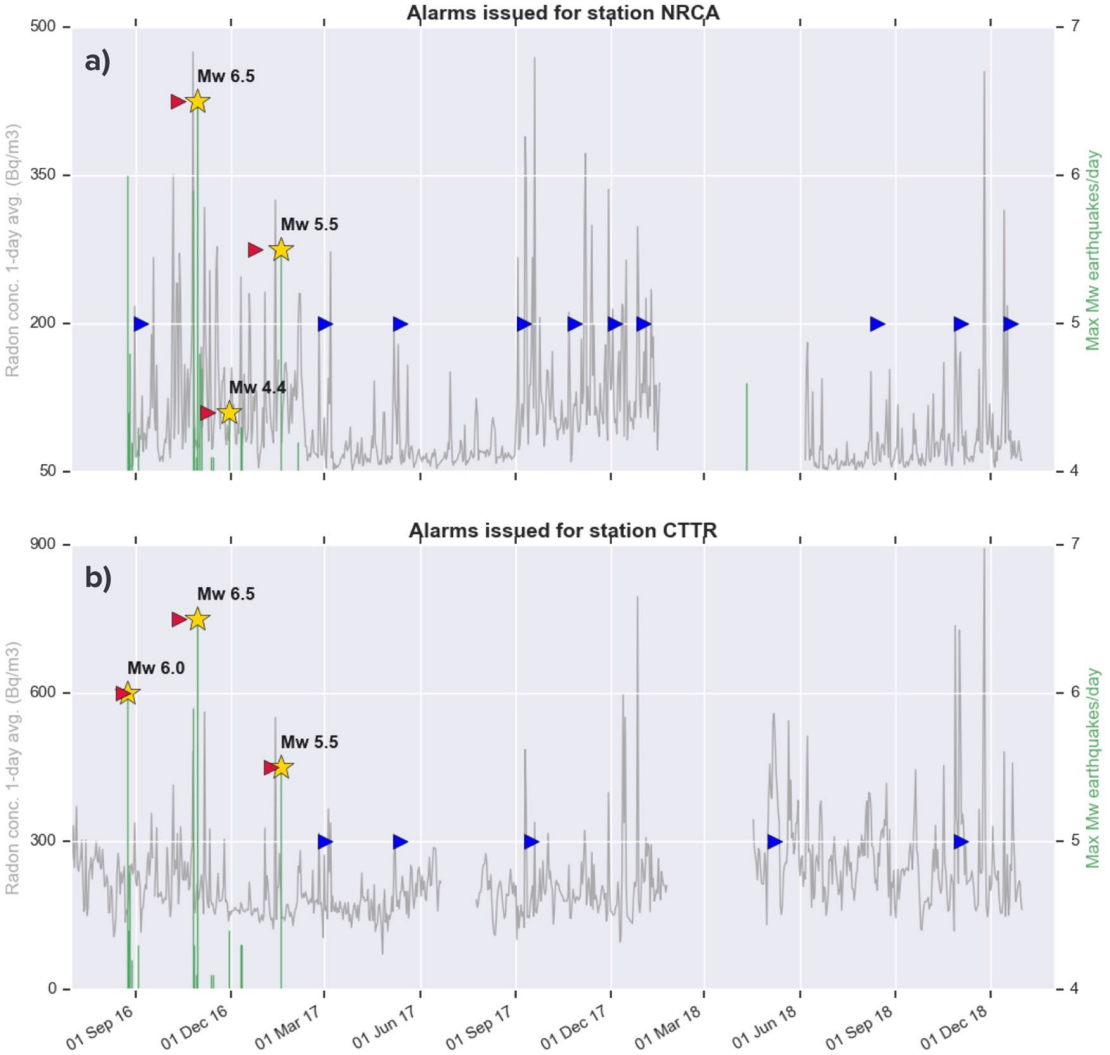
Same as previous figure, with the detection algorithm applied to the rain-corrected time series of radon concentration of NRCA and CTTR station.

Figure 8 is analogous to Fig. 6, with the difference that radon data are corrected for the effect of rain. The DA applied to the NRCA time series successfully forecasts the same events detected on the corresponding raw series, but issuing as many as 10 false alarms. The same behaviour is observed on the rainfall-corrected CTTR times series: the DA not only succeeds in detecting EQ1 and EQ3, but also delivers a further alarm for EQ4. The time advance of the alarm for EQ3 is about 2 weeks, consistent with that observed in Figs. 3 and 6. On the flip side, the number of false alarms throughout 3 years rises from 2 to 5, while on the whole 7-year-long time series its increase is of 6 times. We interpret this result as a consequence of the correction employed, which amplifies the values of radon concentration, leading the algorithm to detect anomalous radon variations more easily and more often. To get reliable results an ad-hoc tuning of the input parameters for the series corrected is unavoidable.

### Change point analysis and cross-correlation

We applied to NRCA time series an algorithm originally developed in the realm of Earth’s climate system studies that calculates, by means of a Bayesian approach, the posterior probability of multiple change points (CP hereinafter) in a generic climatic time series^27^. The algorithm is able to identify an arbitrary number of CPs in time series, being the maximum number of allowed CPs and the minimum distance between adjacent CPs the input parameters of the algorithm (kmax and dmin, respectively).

**Figure 9:**
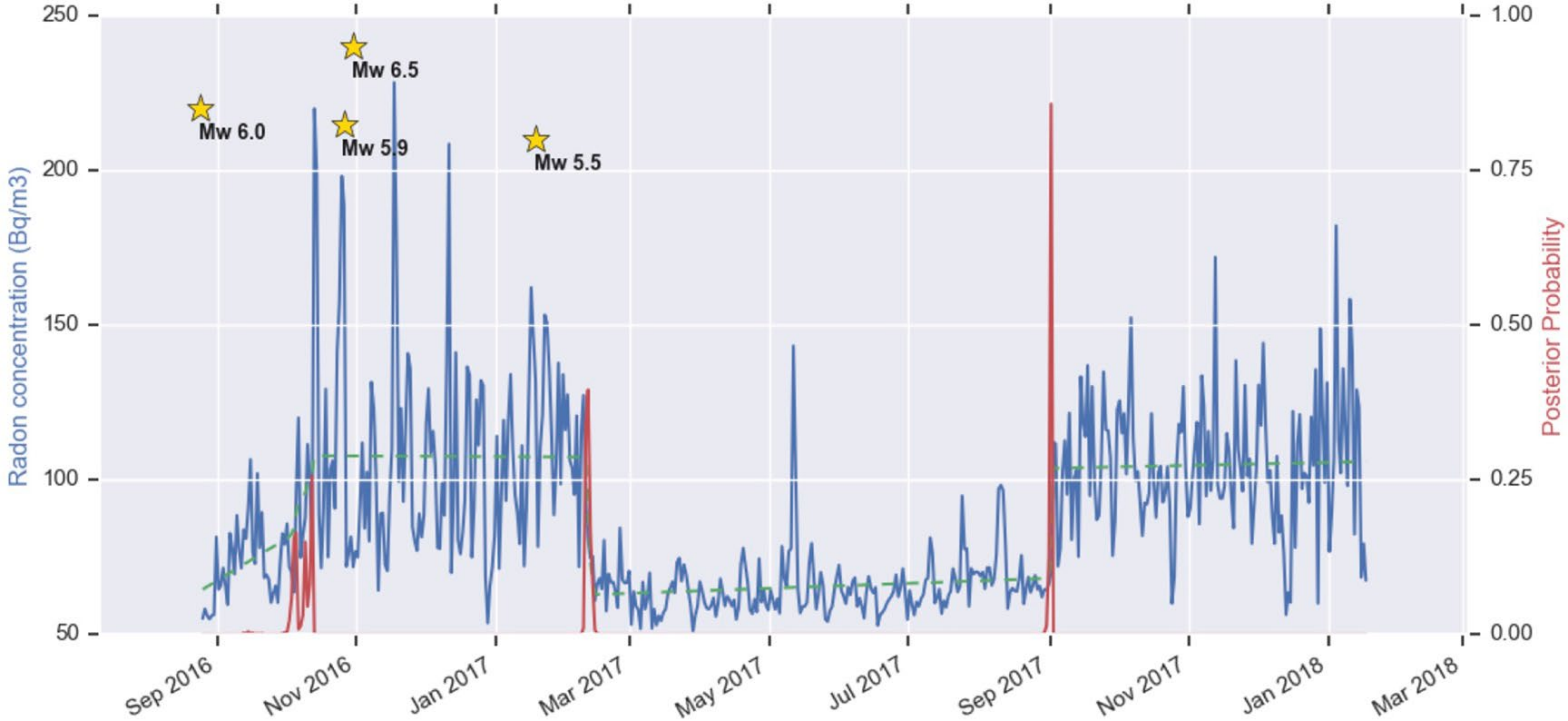
Change point analysis of the time series of radon concentration at NRCA. The blue line represents the daily radon concentration at NRCA, while the green dashed line represents the model predicted by the Bayesian Change Point algorithm. The red line indicates the probability of a CP at each time. The occurrence of the Mw ≥ 5.5 earthquakes in the selected period is marked with yellow stars. The maximum values of probability of a CP occur on October 12, 2016, February 9, 2017 and September 1, 2017.

Figure 9 shows the results of the Bayesian CP algorithm applied to NRCA time series with kmax = 3 and dmin = 2 with a number of sampled solutions fixed to 10,000; repeating the analysis with kmax and dmin in a reasonable range of values obtains similar conclusions. The algorithm finds a CP on 12 October 2016, i.e. preceding EQ3 of about 2 weeks, in agreement with what observed on raw data and what resulted from the DA analysis. The next 2 CPs found have higher a posteriori probability and likely correspond to the abrupt variations of radon emanation which reflect seasonal periodicity. This is observed also for the few CPs detected conducting the Bayesian analysis on the CTTR time series, not shown here for the sake of brevity.

To further check the delay time between variations in radon concentration and seismic activity, we cross-correlate the time series of radon data with the amount of seismic moment (M0) released every day by the earthquakes occurred on that day within a radius of 40 km from the station considered. M0 is obtained converting the total moment magnitude (Mw)^28^.

**Figure 10:**
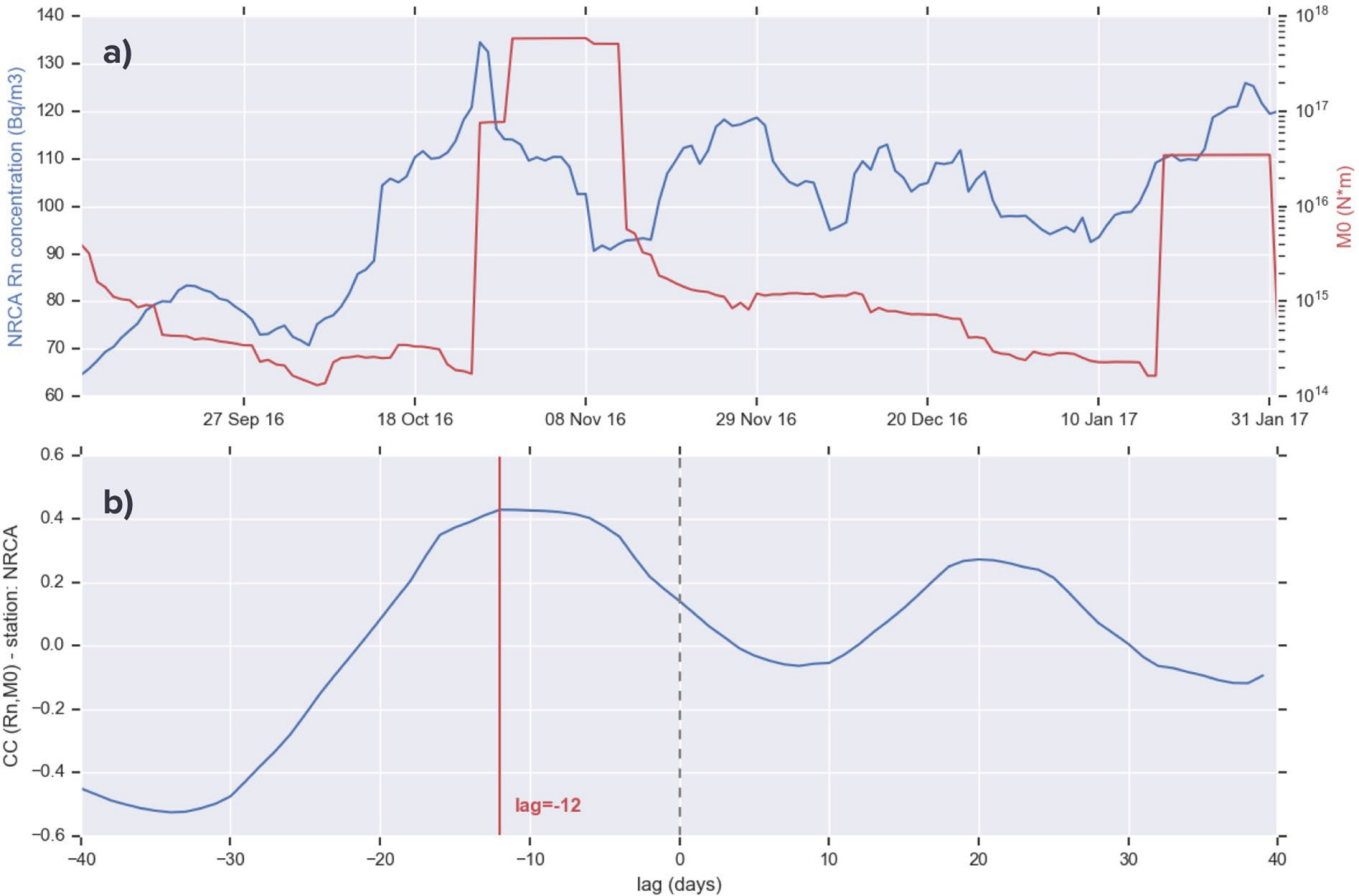
(**a**) 14-day moving-averaged time series of radon concentration at station NRCA (blue) and of seismic moment release M0 (red). (**b**) Cross-correlation between the time series above.

The time lagged correlation between the 14-day moving average of radon concentration and seismic moment release (Fig. 10) results maximum for a lag of -12 days. The negative offset suggests that radon is leading the interaction: correlation is maximized when M0 is pulled backward by 12 frames. The delay time is again in agreement with the time advance resulting from the DA and from the previous analyses.

The plot of the cross-correlation radon-seismic moment obtained for station CTTR (Supplementary Fig. S3) is maximized for a time lag of -8 days, which would be in agreement with the advance time of the alarm preceding the Mw6.5 mainshock (EQ3). But since the DA found two alarms (one before EQ1 and one before EQ3), we cannot attribute this delay to one or the other earthquake without the risk of over interpreting the result.

## Discussion

The comparison of radon concentration time series recorded by two neighboring stations allows to identify a major radon peak about 2 weeks before the largest mainshock on October 30, 2016 (EQ3), shared by both the stations considered, and more pronounced on the station (NRCA), the nearest to the epicentre.

The DA applied to the time series of this station successfully forecasts EQ3, with a time advance of 11 days, consistent with the peak observed on the radon data.

The alarm is also confirmed by a change point analysis, which finds a CP on October 12, 2016, about 2 weeks before EQ3, and by the cross-correlation between radon concentration and seismic moment release, which is maximum for a time lag of -12 days (the negative offset suggests that radon is leading the interaction).

We cannot rule out the hypothesis that the success in forecasting EQ3 may be due to the fact that the positive radon anomaly detected on NRCA time series coincides with the expected seasonal increase in radon concentration. However, when it comes to following years, no alarm is issued, not even during the’ favourable’ season; furthermore, ‘false alarms’ are generally issued in the initial or final phase of the season of maximum radon increase, not during it. Finally, the shape of the radon signal—remarkably similar for the two stations NRCA and CTTR—goes in the direction of excluding a pure seasonal effect even if, given the considerable influence of temperature on soil radon variations, the hypothesis of the environmental engine as their main source cannot be disregarded.

Our forecasting results are even more satisfactory bearing in mind that the detection algorithm was designed not for the radon data of the central Italy stations but for those collected by some stations in southern Italy, in relation to the Pollino seismic sequence of 2012 (where it turned out to be successful)^22^.

On the CTTR time series, despite the lower signal-to-noise ratio, the DA succeeds in forecasting both EQ1, smaller than EQ3 but closer to this station, and EQ3, occurred 24 km away, suggesting that the signal of this mainshock on radon emanation is a strong and robust feature. Thanks to the length of the time series recorded by CTTR (about 7 years), we could test the performance of the DA on the long term. The number of false alarms achieved is about one every two years: a significant result in view of a potential future operational forecast application. Furthermore, most of the false alarms are delivered when the ‘warm’ season starts or ends, indicating a seasonal character that could in principle be removed from the radon data before running the DA. Unfortunately, since radon signals of tectonic and meteorological origin have similar temporal behaviour and similar magnitude, this method has the disadvantage of almost cancelling out also the tectonic signal we want to extract.

Applying an empirical correction for the effect of precipitation confirms the success of DA in forecasting EQ1 on the radon time series of the station CTTR and EQ3 on both stations. This is encouraging, even if we obtain a higher number of false alarms for the corrected time series. Nevertheless, we have to remind that there are strong indications from multi-stations, multi-variate analyses^26^ that local site effects play a major role in modulating radon signal interactions with environmental parameters variation. Such evidence prevents us from the possibility to univocally assess the meteorological correction parameters and, consequently, the results of our approach to deal with atmospheric conditions should be considered as a complementary attempt to give further insights on our dataset and not necessarily more reliable or robust.

Once again this corroborates the importance of deploying a dense network of permanent radon instruments since long time series are helpful to fully appreciate seasonal and meteorological effects. Indeed, we believe that analyses of time series which explore only a limited time window around an hypothetical seismotectonic signal might very likely lead to weak and possibly biased conclusions. On the other hand, data from multiple stations are crucial even when the clarity of signal is poor. Indeed, the very interesting aspect of retrieving common features on data recorded by two stations is the indication that impulsive transients in radon concentration signal could be the expression of a single phenomenon taking place at depth in the crust, and the ability to discern them from the ones originated in the very near field of the instrument.

## Supplementary information

Supplementary information

## Data Availability

The datasets analysed in the current study are available from the corresponding author on reasonable request.
