# Supplementary material for: Monitoring soil radon during the 2016–2017 central Italy sequence in light of seismicity
Source: Sci Rep. 2020 Aug 4;10:13137. doi: 10.1038/s41598-020-69821-2 (PMC7403152; doi:10.1038/s41598-020-69821-2)
Supplement: Supplementary file 1 — Supplementary information [file 41598_2020_69821_MOESM1_ESM.pdf]

Monitoring soil radon during the 2016-2017 central Italy sequence in light of seismicity

Gaia Soldati [1,*], Valentina Cannelli [1], and Antonio Piersanti [1]

[1] Istituto Nazionale di Geofisica e Vulcanologia, via di Vigna Murata 605, 00143, Roma, Italy

corresponding author: gaia.soldati@ingv.it

Supplementary Information

input parameter	NRCA	CTTR
p1	4	4
p2	2.0	1.6
p3	0.7	0.7
p4	1.5	1.5
p5	1.3	1.3

Supplementary Table S1. Values of the selected input parameters for the Detection Algorithm (see Figures)

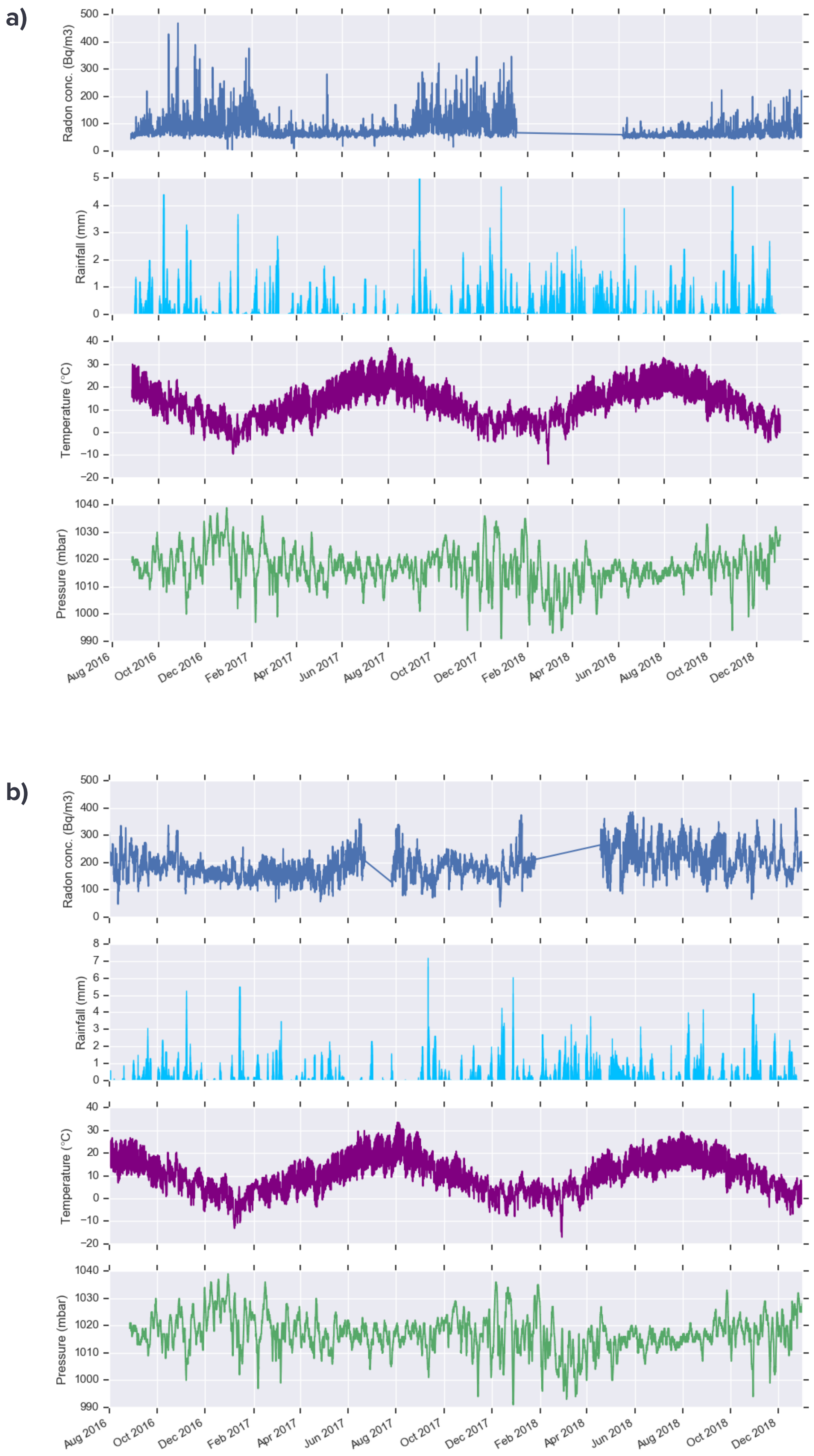

Supplementary Figure S2. Continuous time series of the parameters measured during the years 2016-2018 at station NRCA (a) and 2012-2019 at station CTTR (b): radon concentration, atmospheric pressure, air temperature, precipitation.

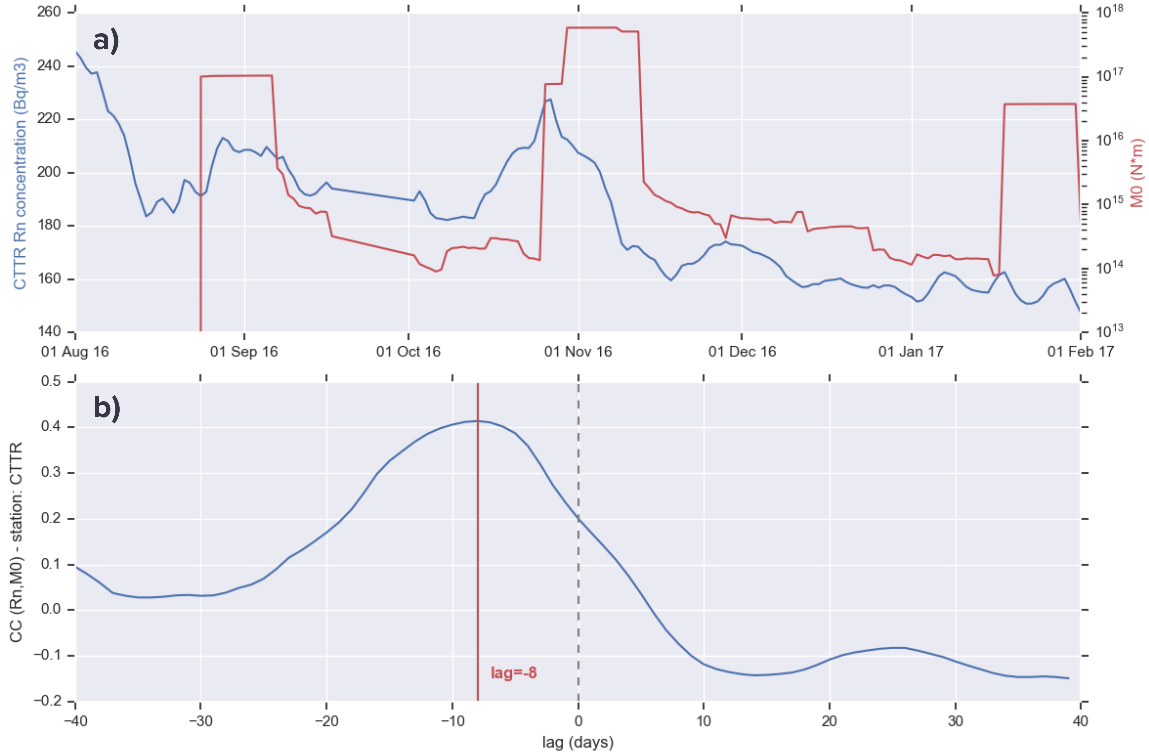

Supplementary Figure S3. a) 14-day moving-averaged time series of radon concentration at station CTTR (blue) and of seismic moment release M0 (red). b) Cross-correlation between the time series above.
